# Interoception, somatic symptoms, and somatization tendency in Chinese individuals with subsyndromal depression: A follow‐up study

**DOI:** 10.1002/pchj.739

**Published:** 2024-02-16

**Authors:** Xiaolu Zhou, Fen Ren, Simon S. Y. Lui, Raymond C. K. Chan

**Affiliations:** ^1^ Neuropsychology and Applied Cognitive Neuroscience Laboratory, CAS Key Laboratory of Mental Health, Institute of Psychology Chinese Academy of Sciences Beijing China; ^2^ College of Education Shanghai Normal University Shanghai China; ^3^ School of Education and Psychology University of Jinan Jinan China; ^4^ Department of Psychiatry, School of Clinical Medicine The University of Hong Kong Hong Kong China; ^5^ Department of Psychology University of Chinese Academy of Sciences Beijing China

**Keywords:** Interoception, schema of somatic focus, somatic symptoms, subsyndromal depression

## Abstract

Interoception refers to the sensation and perception of internal bodily sensations, and may be related to depressive symptoms. Schemata concerning the body vary across different cultures and may influence interoception and symptom presentations of depression. This study explored the relationship between interoception, depressive symptoms, and schema of somatic focus in Chinese people with subsyndromal depression. Thirty‐nine individuals with subsyndromal depression (SD) and 40 healthy controls (HCs) were assessed at baseline and after 3 months. Participants completed the self‐report questionnaires for assessing interoceptive sensibility, somatic and psychological symptoms of depression, and somatization tendency. They also completed the heartbeat perception behavioral task for estimating interoceptive accuracy. The results showed that both the SD and the HC groups showed similar interoceptive accuracy, although the SD group showed heightened interoceptive sensibility. The discrepancy between interoceptive sensibility and interoceptive accuracy is termed the interoceptive trait prediction error (ITPE). The ITPE was positive in SD participants but was negative in HCs. In the entire sample, interoceptive sensibility and the ITPE were correlated with somatic symptoms rather than with psychological symptoms of depression. Interoceptive sensibility partially mediated the relationship between somatization tendency and somatic symptoms, after controlling for psychological symptoms of depression. These results remained stable after 3 months. The shortcomings of the present study were a lack of clinical interview to ascertain diagnosis and a short follow‐up duration. In conclusion, our study suggests that altered interoception occurs in subsyndromal depression. Interoception is related to somatic symptoms of depression. The schema of body was related to depressive symptoms, partially through interoception, in Chinese people with subsyndromal depression.

## INTRODUCTION

Depression commonly involves somatic symptoms (Diagnostic and Statistical Manual of Mental Disorders [DSM‐5]; American Psychiatric Association [APA], 2013), such as sleep disturbance, changes in appetite and/or weight, fatigue, dizziness, aches, and pain (Zhao et al., 2018). Depressive patients' awareness and presentations of these somatic symptoms, that is, “presenting somatization” (Kirmayer & Robbins, 1991), vary across different cultures (Simon et al., 1999). Chinese patients are more likely than Europeans to present with somatic symptoms when they become depressed (Kleinman, 1982, 1986; Parker et al., 2001; Ryder et al., 2008). The “somatization tendency” in the Chinese population may be related to distinct cognitive schemata affecting interoception (Craig, 2002; Zhou et al., 2016).

Interoception refers to the sensation and interpretation of afferent signals from internal body states (Khalsa et al., 2018; Khalsa & Lapidus, 2016). Interoception can involve accuracy and sensibility facets. Interoceptive accuracy refers to the ability to precisely monitor actual physiological changes (Khalsa et al., 2018) and is typically operationalized as the degree of accuracy in detecting bodily signals (such as heartbeat; Schandry, 1981). In contrast, interoceptive sensibility refers to the ability to the extent to attend or perceived internal body states (Chentsova‐Dutton & Dzokoto, 2014) and is typically operationalized as the reported frequency of a variety of bodily sensations on self‐report questionnaires (e.g., the Body Perception Questionnaire [BPQ]; Porges, 1993). Previous studies examining cultural differences in interoception revealed a striking paradox, namely that East Asians and West Africans reported higher levels of interoceptive sensibility, but lower levels of interoceptive accuracy, relative to European Americans (Chentsova‐Dutton & Dzokoto, 2014; Dzokoto, 2010; Ma‐Kellams et al., 2012). Various mechanisms have been proposed to explain such cultural differences, and one possible reason may be related to the culture‐specific schemata of somatic focus commonly held among East Asians and West Africans, which may heighten their interoceptive sensibility but undermine their interoceptive accuracy (Chentsova‐Dutton & Dzokoto, 2014).

Recent theoretical models link depression to impaired interoception (Harshaw, 2015; Paulus & Stein, 2010). Using the heartbeat perception task (Schandry, 1981), previous studies have reported decreased interoceptive accuracy in moderately depressed individuals compared with in healthy people (Furman et al., 2013; Terhaar et al., 2012), but severely depressed individuals showed intact interoceptive accuracy in detecting heartbeat (Dunn et al., 2007). Research on the relationship between interoceptive sensibility and depression is more consistent, suggesting impairments of interoceptive sensibility in people with depression (Eggart & Valdés‐Stauber, 2021; Ewing et al., 2017; Fissler et al., 2016; Wiebking et al., 2010). Paulus and Stein (2010) proposed that depression involves a “dysfunctional interoceptive state” with heightened discrepancies between perceived and actual bodily signals. This state is operationalized as the difference between interoceptive sensibility and interoceptive accuracy and termed the “interoceptive trait prediction error (ITPE)” (Garfinkel et al., 2016). However, the postulation of the ITPE has yet to be examined empirically in people with depression.

The extant literature on interoception and depression has several shortcomings. First, few studies have utilized culturally diverse samples other than Westerners, and very few have utilized samples with subsyndromal depression. Second, few studies have examined the relationship between interoception and the symptomatology of depression, although a recent conceptualization described the relationship between altered interoception and somatic symptoms (Harshaw, 2015). Third, no study to date has directly examined the complex relationship between interoception, specific symptoms of depression, and cultural factors. Lastly, no study in this area has adopted a follow‐up design to clarify the naturalistic evolution and stability of the relationship between interoception, specific symptoms of depression, and cultural factors.

Therefore, this study aimed to examine (1) interoception (accuracy, sensibility, and the ITPE) in Chinese people with subsyndromal depression; (2) the relationship between interoception and somatic/psychological symptoms of depression; (3) the role of culture‐bound cognitive schema of somatization tendency in interoception and depressive symptoms; and (4) the 3‐month stability of such a complex relationship. Besides the conceptual model proposed by Harshaw (2015), recent findings have suggested that interoceptive sensibility is associated with somatic symptoms but not with psychological symptoms of depression among Chinese people (Wang et al., 2020). Therefore, we hypothesized that (1) individuals with subsyndromal depression would show altered interoceptive accuracy, interoceptive sensibility, and ITPE, relative to HCs; (2) interoceptive accuracy, interoceptive sensibility, and ITPE would be correlated with somatic symptoms rather than with psychological symptoms of depression; (3) the relationship between somatization tendency and somatic symptoms would be mediated by interoception; and (4) such a relationship would be stable over a 3‐month period.

## METHODS

### Participants

**FIGURE 1:**
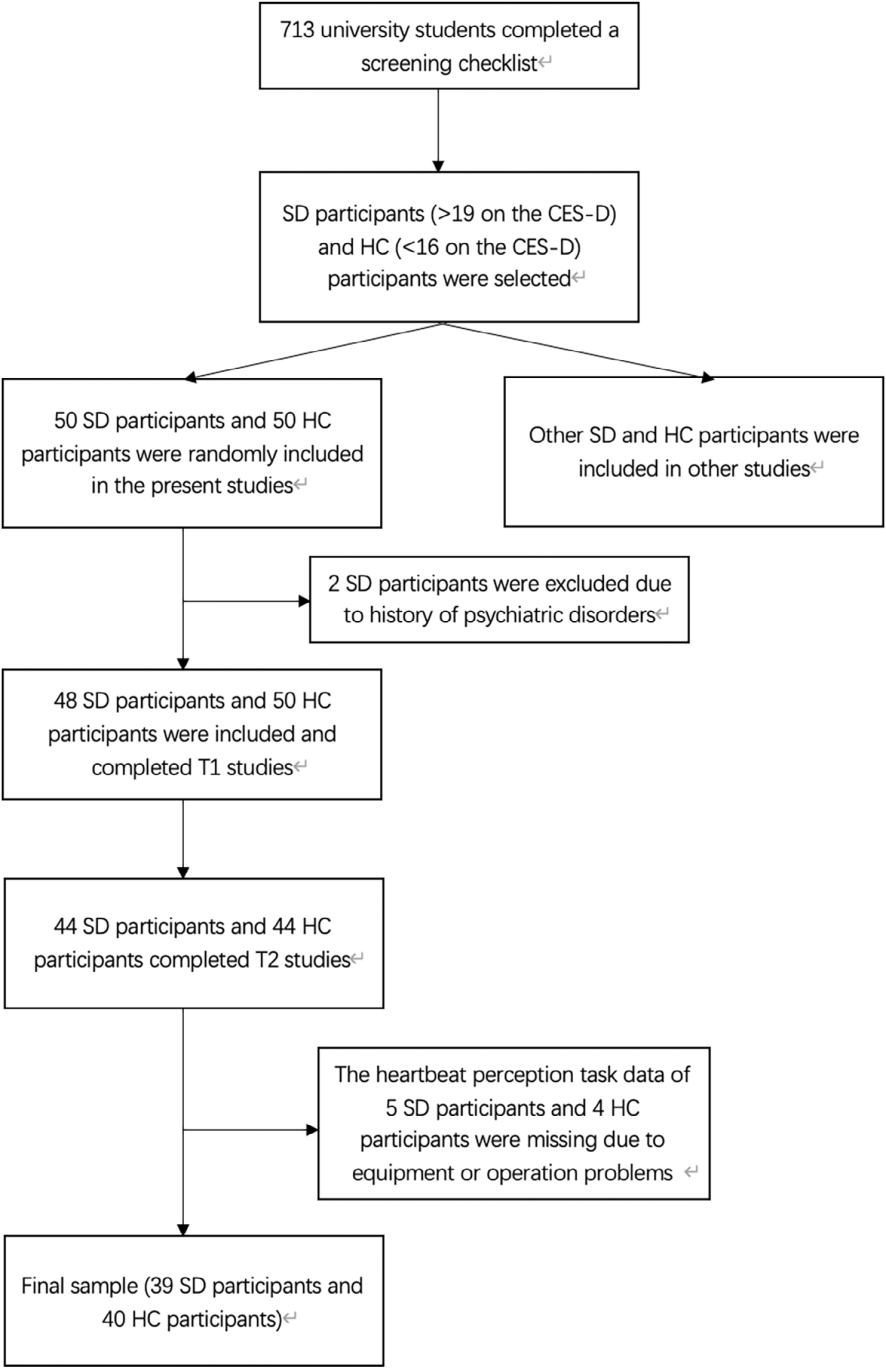
Flow chart of the inclusion and exclusion process of participants in the current study.

Participants were recruited from a university in East China via website postings. A pool of university students (*n* = 713) completed a screening checklist (including simple demographic questions and the Centre for Epidemiological Studies Depression Scale [CES‐D]; Radloff, 1977) for eligibility criteria. Eligible participants were categorized into the subsyndromal depression (SD) group if they scored more than 19 (“have depressive symptoms”; Zhang, 1998) on the CES‐D. Participants were deemed to belong to the healthy control (HC) group if they scored below 16 (“no depressive symptom”; Zhang, 1998) on the CES‐D. The university students who scored 16–19 on the CES‐D were excluded because their depressive symptoms were considered “borderline” (Zhang, 1998). The exclusion criteria included (1) a history of psychiatric disorders, and (2) receiving psychotropic medications at the time of data collection. Figure 1 illustrates the subject recruitment procedures and criteria. Our final sample comprised 39 SD participants (11 male) and 40 HC participants (11 male).

### Measures

#### Interoceptive accuracy

The heartbeat perception task (Schandry, 1981) was used to measure interoceptive accuracy. Participants were instructed to silently count their heart beats during four time intervals of 25, 35, 45, and 100 s, presented in a fixed order. After each time block, participants were required to verbally report the number of heartbeats they had counted to the experimenter. The actual cardiovascular activities during each time interval were recorded using an integrated system (Biopac MP150 electrocardiogram amplifier, Acqknowlege 4.4 software, Biopac Systems, Inc., Goleta, CA, USA). Interoceptive accuracy scores were calculated as the mean scores across the four time intervals according to the formula (Garfinkel et al., 2015): 1 − (|nbeats_real_ − nbeats_reported_|)/([nbeats_real_ + nbeats_reported_]/2).

#### Interoceptive sensibility

The body awareness subscale of the Body Perception Questionnaire—Short Form (BPQ‐SF‐BA; Cabrera et al., 2018) was used to assess interoceptive sensibility. This subscale has 26 items for assessing bodily sensations (e.g., goose bumps) on a 5‐point Likert scale (1 = “*never*”; 5 = “*always*”). High scores on this subscale reflect hypersensitivity, and low scores reflect hyposensitivity. This subscale had good psychometric properties among Chinese college students (Wang et al., 2020). Its internal consistency in the current study was excellent (T1: Cronbach's α = .93; T2: Cronbach's α = .95).

#### ITPE

The ITPE score (Garfinkel et al., 2016) for each participant was calculated as the difference between *z* scores of the BPQ‐SF‐BA and interoceptive accuracy. A positive ITPE value indicates that an individual tends to overestimate his/her interoceptive ability, whilst a negative value indicates a tendency to underestimate interoceptive ability.

#### Somatic and psychological symptoms of depression

The somatic symptom subscale (11‐item) and the psychological symptom subscale (17‐item) of the questionnaire developed by Ryder et al. (2008) were used to assess somatic/psychological depressive symptoms. These two subscales were derived from the CES‐D, the 30‐item General Health Questionnaire (Goldberg, 1972; Goldberg & Williams, 1988), and the 30‐item Chinese Health Questionnaire (Cheng & Williams, 1986). All items are rated on a 0–3 rating scale from “*rarely or none of the time*” to “*most of the time*.” The two subscales can differentiate somatic from psychological symptoms in the Chinese setting (Ryder et al., 2008). In this study, internal consistencies of the somatic (T1: Cronbach's α = .92; T2: Cronbach's α = .88) and the psychological (T1: Cronbach's α = .96; T2: Cronbach's α = .95) symptom subscales were good.

#### Somatization tendency

The somatization subscale of the Cross‐Cultural (Chinese) Personality Assessment Inventory (CPAI‐2‐S; Cheung et al., 2008) was used to assess somatization tendency. This subscale is a 16‐item measure for measuring beliefs and bodily responses that may increase the tendency to emphasize somatic sensations when individuals become distressed (e.g., when I am not happy, I would not say so; I would only get a headache or feel tired), using a 5‐point Likert scale (1 = “*not at all characteristic or true of me*”; 5 = “*extremely characteristic or true of me*”). The internal consistency was good (T1: Cronbach's α = .90; T2: Cronbach's α = .89).

### Procedure

Online recruitment was conducted in July 2021. Eligible participants were invited to attend the first‐wave data collection (T1, September 2021). Participants completed the online questionnaires before performing the heartbeat interoception task. The same sample was invited to provide the second‐wave data (T2) after 3 months (December 2021), by completing the same set of questionnaires and experimental task.

This study was approved by the Ethics Committees at the Institute of Psychology, Chinese Academy of Sciences (Protocol number: H20047) and Shanghai Normal University (Protocol number: SHNU Ethics [2021] no. 10), and it was conducted in accordance with the Helsinki Declaration, 2013 revision. All participants provided written informed consent. Each participant was given US$9 per hour as incentives upon completion of the assessments.

### Data analysis

Group differences in interoceptive accuracy, interoceptive sensibility, ITPE, somatic symptoms of depression, psychological symptoms of depression, and somatization tendency at T1 and T2 were examined using repeated measures analysis of covariance (ANCOVAs). Age was entered as a covariate, because the SD group was younger than the HC group. Given that the somatic symptom subscale and psychological symptom subscale were significantly correlated (*r*_T1_ = .76, *p* = .000; *r*_T2_ = .78, *p* = .000), we used partial correlations to determine the relationship between interoception, somatic symptoms, and psychological symptoms of depression, while controlling for the other depressive symptom subscales at each time point (T1/T2). The partial correlations were conducted using the entire sample. For mediation analysis, the PROCESS macro of SPSS (IBM, Amunk City, NY, USA) was used. In the mediation models at each time point, somatization tendency was set as the independent variable, interoception (accuracy, sensibility, and ITPE) as mediators, somatic symptoms of depression as the outcome variable, and psychological symptoms of depression as the control variable. The number of bootstrapped sampling was set at 5000. Our sample size satisfied the minimum subject number required for the mediation model, that is, 76 (effect size *f* = .39, α = .05, power = .80). Regression slope difference tests were conducted to compare the corresponding coefficients of the two mediation models.

## RESULTS

### Study sample

The HC group was older in age than the SD group, *M*_HC_ = 22.57, *SD*_HC_ = 2.64, *M*_SD_ = 20.87, *SD*_SD_ = 2.11, *t*(74) = 3.10, *p* = .00, *d* = .71. The two groups were comparable in gender ratio (*χ* ^2^_(1)_ = .43, *p* = .52), body mass index (BMI) [*M*_HC_ = 22.21, *SD*_HC_ = 5.68, *M*_SD_ = 20.86, *SD*_SD_ = 3.31, *F*(1, 73) = 2.87, *p* = .10, partial *η* ^2^ = .04], and heart rate during the heartbeat perception tasks at the two time‐points [*F*(1, 73) = .46, *p* = .50, partial *η* ^2^ = .01].

### Group comparisons in interoception

Descriptive data are presented in Table 1. For interoceptive accuracy, we did not find any significant main effect of Group [*F*(1, 73) = .28, *p* = .60, partial *η* ^2^ = .00], main effect of Time‐point [Greenhouse–Geisser‐adjusted *F*(1, 73) = .02, *p* = .89, partial *η* ^2^ = .00], main effect of Age [*F*(1, 73) = .02, *p* = .89, partial *η* ^2^ = .00], interaction effect between Group and Time‐point [Greenhouse–Geisser‐adjusted *F*(1, 73) = .01, *p* = .94, partial *η* ^2^ = .00], and interaction effect between Age and Time‐point [Greenhouse–Geisser‐adjusted *F*(1, 73) = .01, *p* = .93, partial *η* ^2^ = .00].

**TABLE 1:** Descriptive statistics of the study variables.

	Healthy controls		Subsyndromal depression	
	T1	T2	T1	T2
Accuracy	.65 (.29)	.63 (.38)	.69 (.31)	.67 (.28)
BPQ‐SF‐BA	46.57 (10.81)	42.54 (11.62)	65.18 (14.50)	61.16 (15.33)
ITPE	−.55 (1.24)	−.51 (1.23)	.46 (1.39)	.52 (1.03)
SOM	2.08 (2.71)	1.43 (2.28)	9.37 (6.95)	7.58 (5.33)
PSY	4.00 (6.03)	4.51 (6.59)	22.03 (8.96)	17.39 (10.08)
CPAI‐2‐S	23.08 (5.88)	21.24 (4.53)	38.74 (10.52)	32.92 (8.98)

*Note*: BPQ‐SF‐BA = Body Awareness Subscale of the Body Perception Questionnaire—Short Form; ITPE = interoceptive trait prediction error; SOM = somatic symptoms of depression subscale; PSY = psychological symptoms of depression subscale; CPAI‐2‐S = somatization subscale of the Cross‐Cultural (Chinese) Personality Assessment Inventory.

For interoceptive sensibility, the SD group scored significantly higher on the BPQ‐SF‐BA: *F*(1, 72) = 51.69, *p* = .000, partial *η* ^2^ = .42, *ω* ^2^ = .33. However, the main effect of Time‐point [Greenhouse–Geisser‐adjusted *F*(1, 72) = .69, *p* = .41, partial *η* ^2^ = .01], the main effect of Age [*F*(1, 72) = 1.25, *p* = .27, partial *η* ^2^ = .02], the interaction effect between Group and Time‐point [Greenhouse–Geisser‐adjusted *F*(1, 72) = .13, *p* = .72, partial *η* ^2^ = .00], and the interaction effect between Age and Time‐point [Greenhouse–Geisser‐adjusted *F*(1, 72) = 1.22, *p* = .27, partial *η* ^2^ = .02] all failed to reach statistical significance.

Whilst the SD group showed a positive ITPE value, and thus their interoceptive sensibility outperformed their interoceptive accuracy, the HC group showed a negative ITPE value, and thus their interoceptive accuracy outperformed their interoceptive sensibility. The two groups differed significantly in ITPE: *F*(1, 72) = 19.36, *p* = .00, partial *η* ^2^ = .21, *ω* ^2^ = .13. However, ITPE did not change over 3 months: Greenhouse–Geisser‐adjusted *F*(1, 72) = 1.17, *p* = .28, partial *η* ^2^ = .02. The main effect of Age [*F*(1, 72) = 0.88, *p* = .35, partial *η* ^2^ = .01], the interaction effect between Group and Time‐point [Greenhouse–Geisser‐adjusted *F*(1, 72) = .08, *p* = .78, partial *η* ^2^ = .00] and the interaction effect between Age and Time‐point [Greenhouse– Geisser‐adjusted *F*(1, 72) = 1.11, *p* = .30, partial *η* ^2^ = .02] all failed to reach statistical significance.

### Group comparisons in depressive symptoms

As shown in Table 1, the SD group scored significantly higher on somatic symptoms of depression: *F*(1, 72) = 45.75, *p* = .000, partial *η* ^2^ = .39, *ω* ^2^ = .30. We did not find a significant main effect of Time‐point [Greenhouse–Geisser‐adjusted *F*(1, 72) = 1.71, *p* = .20, partial *η* ^2^ = .02], main effect of Age [*F*(1, 72) = .35, *p* = .56, partial *η* ^2^ = .01], interaction effect between Group and Time‐point [Greenhouse–Geisser‐adjusted *F*(1, 72) = 2.40, *p* = .13, partial *η* ^2^ = .03], and interaction effect between Age and Time‐point [Greenhouse–Geisser‐adjusted *F*(1, 72) = 2.46, *p* = .12, partial *η* ^2^ = .03].

Furthermore, the SD group scored higher on psychological symptoms of depression: *F*(1, 72) = 85.17, *p* = .000, partial *η* ^2^ = .54, *ω* ^2^ = .53. We did not find any significant change in the psychological symptom score for the two groups over 3 months [Greenhouse–Geisser‐adjusted *F*(1, 72) = 1.21, *p* = .27, partial *η* ^2^ = .02], and the main effect of Age [*F*(1, 72) = .73, *p* = .40, partial *η* ^2^ = .01]. The interaction effect between group and time was significant: Greenhouse–Geisser‐adjusted *F*(1, 72) = 7.25, *p* = .01, partial *η* ^2^ = .09, *ω* ^2^ = .02. The SD group scored significantly higher at T1 than at T2, *t*(37) = 2.90, *p* = .01, *d* = .47, while the HC group showed stable psychological symptom scores over 3 months, *t*(36) = −.35, *p* = .73, *d* = .06. The interaction effect between Age and Time‐point was not significant: Greenhouse–Geisser‐adjusted *F*(1, 72) = 1.73, *p* = .19, partial *η* ^2^ = .02.

### Group comparison in somatization tendency

The SD group scored significantly higher on the CPAI‐2‐S: *F*(1, 72) = 63.83, *p* = .000, partial *η* ^2^ = .47, *ω* ^2^ = .46. We did not find significant main effect of Time‐point [Greenhouse–Geisser‐adjusted *F*(1, 72) = .05, *p* = .83, partial *η* ^2^ = .00], main effect of Age [*F*(1, 72) = .27, *p* = .61, partial *η* ^2^ = .00]. However, the Group × Time‐point interaction effect was significant: Greenhouse–Geisser‐adjusted *F*(1, 72) = 6.92, *p* = .01, partial *η* ^2^ = .09, *ω* ^2^ = .01. We did not find significant interaction effect between Age and Time‐point: Greenhouse–Geisser‐adjusted *F*(1, 72) = .55, *p* = .46, partial *η* ^2^ = .01.

### Interoception and depressive symptoms

Table 2 shows the results. At T1, somatic symptoms of depression were positively correlated with interoceptive sensibility and ITPE, but not significantly associated with interoceptive accuracy, controlling for psychological symptoms of depression. We did not find any significant correlation of psychological symptoms of depression with interoceptive accuracy, sensibility, and ITPE, after controlling for somatic symptoms of depression. The significant and non‐significant associations were replicated at T2. Specifically, somatic symptoms of depression were significantly related to interoceptive sensibility and ITPE; non‐significant associations were found between psychological symptoms of depression and all facets of interoception.

**TABLE 2:** Partial correlations between interoception and depressive symptoms.

	Accuracy_T1_	BPQ‐SF‐BA _T1_	ITPE _T1_
SOM _T1_	−.01	.45***	.27*
PSY _T1_	.11	.22	.04

	Accuracy_T2_	BPQ‐SF‐BA _T2_	ITPE _T2_
SOM _T2_	.09	.47***	.23*
PSY _T2_	.02	.22	.11

*Note*: T1 = first wave of data; T2 = second wave of data; BPQ‐SF‐BA = Body Awareness Subscale of the Body Perception Questionnaire—Short Form; ITPE = interoceptive trait prediction error; SOM = somatic symptoms of depression subscale; PSY = psychological symptoms of depression subscale.

* *p* < .05;

*** *p* < .001.

### Results of mediation analysis

**FIGURE 2:**
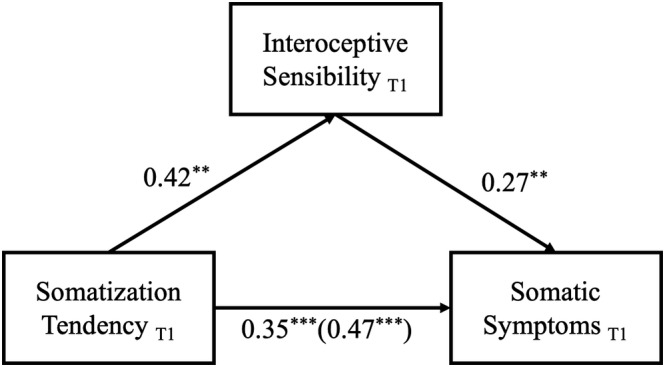
Mediating effect of interoceptive sensibility on somatization tendency and somatic symptoms of depression at T1.

At T1, only interoceptive sensibility was a significant mediator. The total effect of somatization tendency on somatic symptoms of depression was significant, point estimate = .26, 95% confidence interval (CI) = [.15, .37]; the indirect effect of somatization tendency on somatic symptoms of depression through interoceptive sensibility was significant, point estimate = .06, 95% CI = [.02, .12]; the direct effect of somatization tendency on somatic symptoms was also significant, point estimate = .20, 95% CI = [.08, .31]. The direct effect and the indirect effect accounted for 75.88% and 24.12% of the total effect respectively. These results indicate that interoceptive sensibility partially mediated the relationship between somatization tendency and somatic symptoms of depression (see Figure 2, presented with standardized coefficients).

**FIGURE 3:**
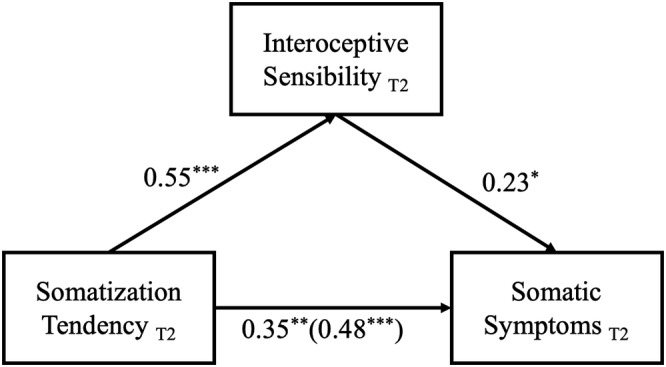
Mediating effect of interoceptive sensibility on somatization tendency and somatic symptoms of depression at T2. * *p* < .05; ** *p* < .01; *** *p* < .001.

The same results were found at T2 (see Figure 3, presented with standardized coefficients). Specifically, interoceptive sensibility was again the only significant mediator. The total effect of somatization tendency on somatic symptoms of depression was significant, point estimate = .27, 95% CI = [.16, .37]; the indirect effect of somatization tendency on somatic symptoms of depression through interoceptive sensibility was significant, point estimate = .07, 95% CI = [.01, .15]; the direct effect of somatization tendency on somatic symptoms was also significant, point estimate = .19, 95% CI = [.08, .31]. The direct effect and the indirect effect accounted for 73.38% and 26.66% of the total effect respectively. Results of the regression slope difference tests showed that, for all the paths of the two mediation models, the regression coefficients were non‐significant (see Table 3). These results further support the notion that interoceptive sensibility partially mediated the relationship between somatization tendency and somatic symptoms of depression.

**TABLE 3:** Coefficient differences of the T1 and T2 mediation models.

	*B*		*SE*		*t*(*df*)	*p*
	T1	T2	T1	T2		
Somatization tendency → Interoceptive sensibility (a)	0.5661	1.0083	0.1708	0.1981	1.691 (150)	0.09
Interoceptive sensibility → Somatic symptoms (b)	0.1098	0.0701	0.0362	0.0284	0.863 (150)	0.39
Somatization tendency→ Somatic symptoms (c′)	0.1957	0.1946	0.0566	0.0566	0.014 (150)	0.99
Somatization tendency→ Somatic symptoms (c)	0.2579	0.2652	0.0557	0.0504	0.097 (150)	0.92

*Note*: *B*, Unstandardized coefficient; *SE*, standard error; (a) relationship between somatization tendency and interoceptive sensibility; (b) relationship between interoceptive sensibility and somatic symptoms when controlling for somatization tendency; (c′) relationship between somatization tendency and somatic symptoms when controlling for interoceptive sensibility (direct effect); (c) relationship between somatization tendency and somatic symptoms (total effect).

## DISCUSSION

This study attempted to examine the relationship between interoception, depressive symptoms, and the schema of the body in Chinese people with subsyndromal depression. The major findings are summarized as follows. (1) Individuals with subsyndromal depression scored higher in interoceptive sensibility, relative to healthy controls. (2) At baseline and after 3 months, interoceptive sensibility was differentially associated with depressive symptoms. Specifically, interoceptive sensibility was stably associated with somatic symptoms of depression, but not with psychological symptoms of depression. (3) Interoceptive sensibility partially mediated the relationship between the schema of the body and somatic symptoms of depression at baseline, and this mediation model remained stable after 3 months.

Our hypotheses regarding interoceptive accuracy were not fully supported by the findings. Specifically, our findings suggested that individuals with subsyndromal depression did not differ from controls in interoceptive accuracy. Furthermore, interoceptive accuracy was not correlated with somatic symptoms nor with psychological symptoms of depression. Although these findings are consistent with Solano López and Moore's (2019) prior findings, in which a non‐significant association was found between depressive mood and interoceptive accuracy in a sample predominated by African Americans, we attempted to account for the non‐significant association between interoceptive accuracy and depressive symptoms in the present sample. Given that the extant literature of cross‐cultural studies (Chentsova‐Dutton & Dzokoto, 2014; Dzokoto, 2010; Ma‐Kellams et al., 2012) generally suggests that West Africans and East Asians report less interoceptive accuracy than European Americans, one possible explanation for our finding is that, in a cultural group with lower levels of heartbeat perception accuracy, the range of interoceptive accuracy was narrow, and this restricted range of scores might have resulted in non‐significant correlations with other variables. In fact, a previous study reported that common somatic symptoms in Chinese people are sleep problems, pre‐verbal physical complaints (i.e., extreme physical distress that is hard to articulate, such as preverbal pain), weight and appetite problems, circulatory system complaints, headache, hyposexuality, gastrointestinal symptom problems, and respiratory system complaints (Zhao et al., 2018). While this study measured only heart perception as interoception, future research could use a composite interoceptive accuracy as proxy, representing more visceral perceptions, especially those closely related to the above‐mentioned symptoms in depression.

Driven by the increased interoceptive sensibility in individuals with subsyndromal depression, people with subsyndromal depression and controls differed in the ITPE. Moreover, the ITPE was positively associated with somatic symptoms of depression. These findings apparently support the important role of “top‐down” beliefs in interoceptive state in the pathogenesis of depression (Paulus & Stein, 2010).

This study has several implications. Our work is one of the first few to empirically examine the relational model involving interoception, depressive symptoms, and the somatic focus schema, namely somatization tendency. Our findings suggest that the somatic focus schema is important for a better understanding of the development of depressive somatic symptoms among Chinese people. The somatic focus values the body and facilitates attention to bodily changes. When an annoying or strange bodily sensation (e.g., a headache) is related to dangerous or disastrous outcomes, this bodily sensation (interoception) may turn into full‐blown symptoms (Ryder et al., 2021). Moreover, future studies could explore other possible top‐down factors shaping interoception and depressive symptoms. Ma‐Kellams et al. (2012) found that the tendency to attend to contextual cues rather than to internal states may explain the differences between East Asians and European Americans in interoceptive accuracy. Ryder et al. (2008) found that the Chinese somatization of depression in comparison with that of European Canadians was related to the tendency to pay attention to external stimuli. As such, future studies could further clarify whether this schema of attention is another factor affecting interoception and depressive symptoms among Chinese people. In addition, this study revealed that altered interoception is present in individuals with subsyndromal depression. Future studies should further examine the replicability of the findings and the utility of interoceptive sensibility as putative targets for preventive interventions. Individuals with subsyndromal depression should be able to be aware of their interoceptive sensations, and future research should investigate whether psychological interventions on one's interoceptive sensations could prevent the development of depressive symptoms. Finally, this study demonstrated stable correlations between interoception and somatic symptoms of depression over a 3‐month period. Medications were unlikely to be confounds to these associations, because our participants did not receive any psychotropic medication. Antidepressant medications can affect one's interoception (Eggart et al., 2019), but it is hard to control for medication effects in samples with clinical depression.

Several limitations should be borne in mind. First, we relied on reports from the participants that they had no history of psychiatric disorders and were not receiving any medication. Future studies should ascertain the clinical diagnosis more stringently using structured clinical interviews. Second, our participants were recruited from a single site and were limited to university students, which affects the generalizability of our findings. Third, we did not include a clinical group for comparison. Moreover, our follow‐up duration was short. Future studies should clarify the long‐term trajectories of the relationship between interoception and depressive symptoms. Lastly, our study design meant we could not infer any causative relationship between interoception, depressive symptoms, and somatization tendency.

To conclude, people with subsyndromal depression exhibit altered interoception, especially interoceptive sensibility. Altered interoceptive sensibility and ITPE were associated with somatic symptoms but not with psychological symptoms of depression. The Chinese schema of the body is related to depressive symptoms, partially through interoception.

## CONFLICT OF INTEREST STATEMENT

The authors declare no conflict of interest.

## ETHICS STATEMENT

This study was approved by the Ethics Committees at Institute of Psychology, Chinese Academy of Sciences (Protocol number: H20047), and Shanghai Normal University (SHNU [2021] protocol number: 10).

## References

[pchj739-bib-0001] American Psychiatric Association . (2013). Diagnostic and statistical manual of mental disorders (5th ed.). American Psychiatric Association.

[pchj739-bib-0002] Cabrera, A. , Kolacz, J. , Pailhez, G. , Bulbena‐Cabre, A. , Bulbena, A. , & Porges, S. W. (2018). Assessing body awareness and autonomic reactivity: Factor structure and psychometric properties of the body perception questionnaire‐short form (BPQ‐SF). International Journal of Methods in Psychiatric Research, 27(2), e1596. 10.1002/mpr.1596 29193423 PMC6877116

[pchj739-bib-0003] Cheng, T. A. , & Williams, P. (1986). The design and development of a screening questionnaire (CHQ) for use in community studies of mental disorders in Taiwan. Psychological Medicine, 16(2), 415–422. 10.1017/s0033291700009247 3726013

[pchj739-bib-0004] Chentsova‐Dutton, Y. E. , & Dzokoto, V. (2014). Listen to your heart: The cultural shaping of interoceptive awareness and accuracy. Emotion, 14(4), 666–678. 10.1037/a0036193 24749640

[pchj739-bib-0005] Cheung, F. M. , Cheung, S. F. , & Leung, F. (2008). Clinical utility of the cross‐cultural (Chinese) personality assessment inventory (CPAI–2) in the assessment of substance use disorders among Chinese men. Psychological Assessment, 20(2), 103–113. 10.1037/1040-3590.20.2.103 18557687

[pchj739-bib-0006] Craig, A. D. (2002). How do you feel? Interoception: The sense of the physiological condition of the body. Nature Reviews Neuroscience, 3(8), 655–666. 10.1038/nrn894 12154366

[pchj739-bib-0007] Dunn, B. D. , Dalgleish, T. , Ogilvie, A. D. , & Lawrence, A. D. (2007). Heartbeat perception in depression. Behaviour Research and Therapy, 45(8), 1921–1930. 10.1016/j.brat.2006.09.008 17087914

[pchj739-bib-0008] Dzokoto, V. A. (2010). Different ways of feeling: Emotion and somatic awareness in Ghanaians and euro‐Americans. Journal of Social, Evolutionary and Cultural Psychology, 4(2), 68–78. 10.1037/h0099299

[pchj739-bib-0009] Eggart, M. , Lange, A. , Binser, M. J. , Queri, S. , & Müller‐Oerlinghausen, B. (2019). Major depressive disorder is associated with impaired interoceptive accuracy: A systematic review. Brain Sciences, 9(6), 131. 10.3390/brainsci9060131 31174264 PMC6627769

[pchj739-bib-0010] Eggart, M. , & Valdés‐Stauber, J. (2021). Can changes in multidimensional self‐reported interoception be considered as outcome predictors in severely depressed patients? A moderation and mediation analysis. Journal of Psychosomatic Research, 141, 110331. 10.1016/j.jpsychores.2020.110331 33338695

[pchj739-bib-0011] Ewing, D. L. , Manassei, M. , Gould van Praag, C. , Philippides, A. O. , Critchley, H. D. , & Garfinkel, S. N. (2017). Sleep and the heart: Interoceptive differences linked to poor experiential sleep quality in anxiety and depression. Biological Psychology, 127, 163–172. 10.1016/j.biopsycho.2017.05.011 28554855 PMC5606300

[pchj739-bib-0012] Fissler, M. , Winnebeck, E. , Schroeter, T. , Gummersbach, M. , Huntenburg, J. M. , Gaertner, M. , & Barnhofer, T. (2016). An investigation of the effects of brief mindfulness training on self‐reported interoceptive awareness, the ability to decenter, and their role in the reduction of depressive symptoms. Mindfulness, 7(5), 1170–1181. 10.1007/s12671-016-0559-z

[pchj739-bib-0013] Furman, D. J. , Waugh, C. E. , Bhattacharjee, K. , Thompson, R. J. , & Gotlib, I. H. (2013). Interoceptive awareness, positive affect, and decision making in major depressive disorder. Journal of Affective Disorders, 151(2), 780–785. 10.1016/j.jad.2013.06.044 23972662 PMC3797260

[pchj739-bib-0014] Garfinkel, S. N. , Seth, A. K. , Barrett, A. B. , Suzuki, K. , & Critchley, H. D. (2015). Knowing your own heart: Distinguishing interoceptive accuracy from interoceptive awareness. Biological Psychology, 104, 65–74. 10.1016/j.biopsycho.2014.11.004 25451381

[pchj739-bib-0015] Garfinkel, S. N. , Tiley, C. , O'Keeffe, S. , Harrison, N. A. , Seth, A. K. , & Critchley, H. D. (2016). Discrepancies between dimensions of interoception in autism: Implications for emotion and anxiety. Biological Psychology, 114, 117–126. 10.1016/j.biopsycho.2015.12.003 26724504

[pchj739-bib-0016] Goldberg, D. P. (1972). The detection of psychiatric illness by questionnaire (Maudsley monograph 21). Oxford University Press.

[pchj739-bib-0017] Goldberg, D. P. , & Williams, P. (1988). A user's guide to the general health questionnaire. NFER‐NELSON.

[pchj739-bib-0018] Harshaw, C. (2015). Interoceptive dysfunction: Toward an integrated framework for understanding somatic and affective disturbance in depression. Psychological Bulletin, 141(2), 311–363. 10.1037/a0038101 25365763 PMC4346391

[pchj739-bib-0019] Khalsa, S. S. , Adolphs, R. , Cameron, O. G. , Critchley, H. D. , Davenport, P. W. , Feinstein, J. S. , Feusner, J. D. , Garfinkel, S. N. , Lane, R. D. , Mehling, W. E. , Meuret, A. E. , Nemeroff, C. B. , Oppenheimer, S. , Petzschner, F. H. , Pollatos, O. , Rhudy, J. L. , Schramm, L. P. , Simmons, W. K. , Stein, M. B. , … Interoception Summit 2016 participants . (2018). Interoception and mental health: A roadmap. Biological Psychiatry. Cognitive Neuroscience and Neuroimaging, 3(6), 501–513. 10.1016/j.bpsc.2017.12.004 29884281 PMC6054486

[pchj739-bib-0020] Khalsa, S. S. , & Lapidus, R. C. (2016). Can interoception improve the pragmatic search for biomarkers in psychiatry. Frontiers in Psychiatry, 7, 121. 10.3389/fpsyt.2016.00121 27504098 PMC4958623

[pchj739-bib-0021] Kirmayer, L. J. , & Robbins, J. M. (1991). Introduction: Concepts of somatization. In L. J. Kirmayer & J. M. Robbins (Eds.), Current concepts of somatization: Research and clinical perspectives (pp. 1–19). American Psychiatric Press.

[pchj739-bib-0022] Kleinman, A. (1982). Neurasthenia and depression: A study of somatization and culture in China. Culture, Medicine, and Psychiatry, 6(2), 117–190. 10.1007/BF00051427 7116909

[pchj739-bib-0023] Kleinman, A. (1986). Social origins of disease and distress: Depression, neurasthenia, and pain in modern China. Yale University Press.

[pchj739-bib-0024] Ma‐Kellams, C. , Blascovich, J. , & McCall, C. (2012). Culture and the body: East—West differences in visceral perception. Journal of Personality and Social Psychology, 102(4), 718–728. 10.1037/a0027010 22309028

[pchj739-bib-0025] Parker, G. , Cheah, Y. C. , & Roy, K. (2001). Do the Chinese somatize depression? A cross‐cultural study. Social Psychiatry and Psychiatric Epidemiology, 36(6), 287–293. 10.1007/s001270170046 11583458

[pchj739-bib-0026] Paulus, M. P. , & Stein, M. B. (2010). Interoception in anxiety and depression. Brain Structure & Function, 214(5–6), 451–463. 10.1007/s00429-010-0258-9 20490545 PMC2886901

[pchj739-bib-0027] Porges, S. (1993). Body perception questionnaire. In Laboratory of Developmental Assessment. University of Maryland.

[pchj739-bib-0028] Ryder, A. G. , Doucerain, M. M. , Zhou, B. , Dere, J. , Jurcik, T. , & Zhou, X. (2021). On dynamic contexts and unstable categories: Steps toward a cultural‐clinical psychology. In M. J. Gelfand , C. Chiu , & Y. Hong (Eds.), Handbook of advances in culture and psychology (pp. 195–243). Oxford University Press.

[pchj739-bib-0029] Ryder, A. G. , Yang, J. , Zhu, X. , Yao, S. , Yi, J. , Heine, S. J. , & Bagby, R. M. (2008). The cultural shaping of depression: Somatic symptoms in China, psychological symptoms in North America? Journal of Abnormal Psychology, 117(2), 300–313. 10.1037/0021-843X.117.2.300 18489206

[pchj739-bib-0030] Schandry, R. (1981). Heart beat perception and emotional experience. Psychophysiology, 18(4), 483–488. 10.1111/j.1469-8986.1981.tb02486.x 7267933

[pchj739-bib-0031] Simon, G. E. , VonKorff, M. , Piccinelli, M. , Fullerton, C. , & Ormel, J. (1999). An international study of the relation between somatic symptoms and depression. The New England Journal of Medicine, 341(18), 1329–1335. 10.1056/NEJM199910283411801 10536124

[pchj739-bib-0032] Solano López, A. L. , & Moore, S. (2019). Dimensions of body‐awareness and depressed mood and anxiety. Western Journal of Nursing Research, 41(6), 834–853. 10.1177/0193945918798374 30178716

[pchj739-bib-0033] Terhaar, J. , Viola, F. C. , Bär, K.‐J. , & Debener, S. (2012). Heartbeat evoked potentials mirror altered body perception in depressed patients. Clinical Neurophysiology, 123(10), 1950–1957. 10.1016/j.clinph.2012.02.086 22541740

[pchj739-bib-0034] Wang, N. , Ren, F. , & Zhou, X. (2020). Factor structure and psychometric properties of the body perception questionnaire‐short form (BPQ‐SF) among Chinese college students. Frontiers in Psychology, 11, 1355. 10.3389/fpsyg.2020.01355 32714241 PMC7344204

[pchj739-bib-0035] Wiebking, C. , Bauer, A. , de Greck, M. , Duncan, N. W. , Tempelmann, C. , & Northoff, G. (2010). Abnormal body perception and neural activity in the insula in depression: An fMRI study of the depressed “material me”. The World Journal of Biological Psychiatry, 11(3), 538–549. 10.3109/15622970903563794 20146653

[pchj739-bib-0036] Zhang, M. (1998). Handbook of rating scales in psychiatry. Hunan Science and Technology Publishing House.

[pchj739-bib-0037] Zhao, D. , Wu, Z. , Zhang, H. , Mellor, D. , Ding, L. , Wu, H. , Wu, C. , Huang, J. , Hong, W. , Peng, D. , & Fang, Y. (2018). Somatic symptoms vary in major depressive disorder in China. Comprehensive Psychiatry, 87, 32–37. 10.1016/j.comppsych.2018.08.013 30195098

[pchj739-bib-0038] Zhou, X. , Peng, Y. , Zhu, X. , Yao, S. , Dere, J. , Chentsova‐Dutton, Y. E. , & Ryder, A. G. (2016). From culture to symptom: Testing a structural model of “Chinese somatization”. Transcultural Psychiatry, 53(1), 3–23. 10.1177/1363461515589708 26076689

